# Respiratory Muscle Strength: New Technology for Easy Assessment

**DOI:** 10.7759/cureus.14803

**Published:** 2021-05-02

**Authors:** Vasileios T Stavrou, Konstantinos N Tourlakopoulos, Zoe Daniil, Konstantinos I Gourgoulianis

**Affiliations:** 1 Laboratory of Cardio-Pulmonary Testing and Pulmonary Rehabilitation, Respiratory Medicine Department, Faculty of Medicine, University of Thessaly, Larissa, GRC

**Keywords:** respiratory muscle strength, athletes, device

## Abstract

Respiratory muscle strength (RMS) is associated with good functionality of the respiratory system. For the general population, it refers to the quality of life, and for the athletes, is related to greater performance. In this study, a comparison was made between two different portable devices, MicroRPM (CareFusion, Kent, United Kingdom) and AirOFit PRO™ (AirOFit, Copenhagen, Denmark), assessing the maximum inspiratory pressure (MIP) and maximum expiratory pressure (MEP). Twenty-one male professional athletes were evaluated on a voluntary basis and randomly used the devices for RMS assessment, while all athletes underwent Pittsburgh Sleep Quality Index (PSQI), pulmonary function tests and ergospirometry. All measurements of MIP and MEP were made with the same methodology and all participants after the efforts answered the question "easy-operation device-information" and dyspnea and/or respiratory fatigue during trials with the CR10 scale. Results showed statistical differences between VO_2max_ and maximal respiratory strength both for AirOFit PRO™ (r=0.526, p=0.014) and in MicroPRM (r=0.567, p=0.007). The PSQI score showed statistical differences in % of predicted values in MEP with the AirOFit PRO™ device (r=0.478, p=0.028). Athletes reported that the AirOFit PRO™ device is easier in operation as a device and provides more information during trial comparisons to MicroPRM (p=0.001). Athletes reported that the AirOFit PRO™ device is easier in operation as a device and provides more information during the trial compared to MicroPRM. The results did not show differences in RMS (MIP and MEP) between devices (p>0.05). For the people who want to train with tele-exercise and/or tele-rehabilitation, the AirOFit PRO™ device would be an important and safe training solution.

## Introduction

Respiratory muscles can become tired and accelerate or aggravate respiratory failure. The strength of respiratory muscles is an indicator of the good functionality of the respiratory system. The low functionality of respiratory muscles is associated with several diseases such as chronic obstructive pulmonary disease, cystic fibrosis, idiopathic pulmonary fibrosis [1] with neurological diseases such as multiple sclerosis [2] and quality of life in the general population [3]. In addition, respiratory muscle strength (RMS) is related to athletic performance [4,5], while a lot of athletes are looking for various techniques to increase the strength of respiratory muscles in order to improve their performance [6].

To improve RMS, it is important to be reliably evaluated [7]. Thus, the purpose of this study was to investigate the maximum inspiratory (MIP) and maximum expiratory pressure (MEP), using two different portable devices (MicroRPM [CareFusion, Kent, United Kingdom] versus AirOFit PRO™ [AirOFit, Copenhagen, Denmark]). We had two hypotheses: If there are differences in processes involved in the devices which could affect the RMS assessment, and if there are differences between devices in parameter "easy-operation device-information."

## Materials and methods

Participants

Twenty-one male professional athletes (runners, n=6; cyclists, n= 11; triathletes, n=4, Table 1) were included in our study on a voluntary basis and randomly (using block randomization) used the devices for RMS assessment (MicroRPM versus AirOFit PRO™). Inclusion criteria were age between ≥20-and ≤50-years-old, training age ≥4 years (≥5 hours per week with HR ≥70 % of max), without recent injury (for the last 12 months) [8], respiratory and/or cardiological disorders [9] and taking any medication and ergospirometry parameters (VE/MVV <85% and TV/IC <85% and Borg scale dyspnea score <5). All volunteers have lived and been trained in less than 100 m altitudes at sea level, for above 10 months [10]. The study was conducted according to the Helsinki declaration for use in Human subjects (No. of Ethical Committee: No. 2800, Scientific Council of University Hospital of Larisa). All the participants gave us their written consent.

**Table 1:** Athletes characteristics. Continuous variables are presented as mean ± standard deviation. FEV1: forced expiratory volume in firsts; FVC: forced vital capacity; fβ: breath frequency; HR: heart rate; IC: inspiratory capacity; PSQI: Pittsburgh Sleep Quality Index; TV: tidal volume; VC: vital capacity; MVV: maximal ventilation volume; VO_2_: oxygen uptake.

	Mean±SD
Age (years)	40.1±7.2
Training age (years)	10.5±4.2
Body mass (kg)	76.2±8.2
Body fat (%)	10.3±1.7
Body mass index (kg/m^2^)	24.9±2.5
Body surface area (m^2^)	1.9±0.2
Lean body mass (%)	72.3±3.0
Total body water (%)	56.8±2.2
FEV_1_, L (% of predicted)	4.6±0.6 (121.6±11.5)
FVC, L (% of predicted)	5.5±0.7 (118.9±12.2)
IC, L (% of predicted)	3.8±0.6 (111.7±20.6)
VC, L (% of predicted)	5.7±0.7 (117.1±11.2)
VO_2max,_ ml/min^−1^/kg^−1^ (% of predicted)	3708.4±678.6 (137.3±21.0)
VCO_2max,_ ml/min^−1^/kg^−1^	4433.9±852.3
HR_max_, bpm^−1^ (% of predicted)	173.4±14.8 (96.4±6.8)
V_E_/MVV	70.7±14.1
TV/IC	78.3±6.5
f_β_, 1/min	42.3±10.1
Borg Scale_Leg fatigue_, score	6.3±1.7
Borg Scale_Dyspnea_, score	4.1±0.5
PSQI score	1.9±1.8

Study protocol

For each athlete, prior to the assessment of the RMS, we recorded anthropometric and morphological characteristics (Table 1) and body composition (Tanita MC-980, Arlington Heights, Illinois, USA), they answered Pittsburgh Sleep Quality Index (PSQI) [8,11], answered medical history questionnaires and underwent pulmonary function test (VIASYS Health Care, Höchberg, Germany) [12] and ergospirometry [13].

Pulmonary function test

All athletes underwent standard spirometry and lung volume measurements, in line with American Thoracic Society (ATS)/European Respiratory Society (ERS) guidelines [11]. Maximal flow-volume loops were conducted for each subject in a sitting position using MasterScreen-CPX pneumotachograph (VIASYS HealthCare). For each pulmonary function test, three maximal flow-volume loops were obtained to determine FVC and FEV1. Thoracic gas volume at inspiratory capacity (IC) and vital capacity (VC) was measured while subjects made gentle pants against the shutter at a rate of <1/s [14].

Ergospirometry

Ergospirometry was performed on an electronic cycle ergometer (Ergoselect 100, Lindenstraße, Germany) Master Screen-CPX and respiratory and cardiac parameters were recorded (VIASYS HealthCare). All athletes, prior to testing, were familiarized with the test via two minutes resting (first stage); for three minutes unloaded cycling as a warm-up (second stage); after the end of the maximal test (third stage), they performed five minutes unloaded cyclings for recovery (fourth stage) purposes. In the third stage, the ramp work rate increased by 20-25 Watts/min until exhaustion was reached [14]. The work rate increment was calculated using the Wasserman et al. [13] formula:

Work rate/min^−^^1 ^(ramp) = (VO_2max_ − VO_2unloaded_)/100; VO_2max_ = (height (cm) − age (years)) × 20;

VO_2unloaded_ = 150 + (6 × Weight(kg))

Moreover, a 12-lead ECG was also employed for HR monitoring (MasterScreen, Hochberg, Germany), while were recorded every two minutes for all phases Borg CR10 scales for leg fatigue and dyspnea [15].

Respiratory muscle strength

MicroRPM

**Figure 1:**
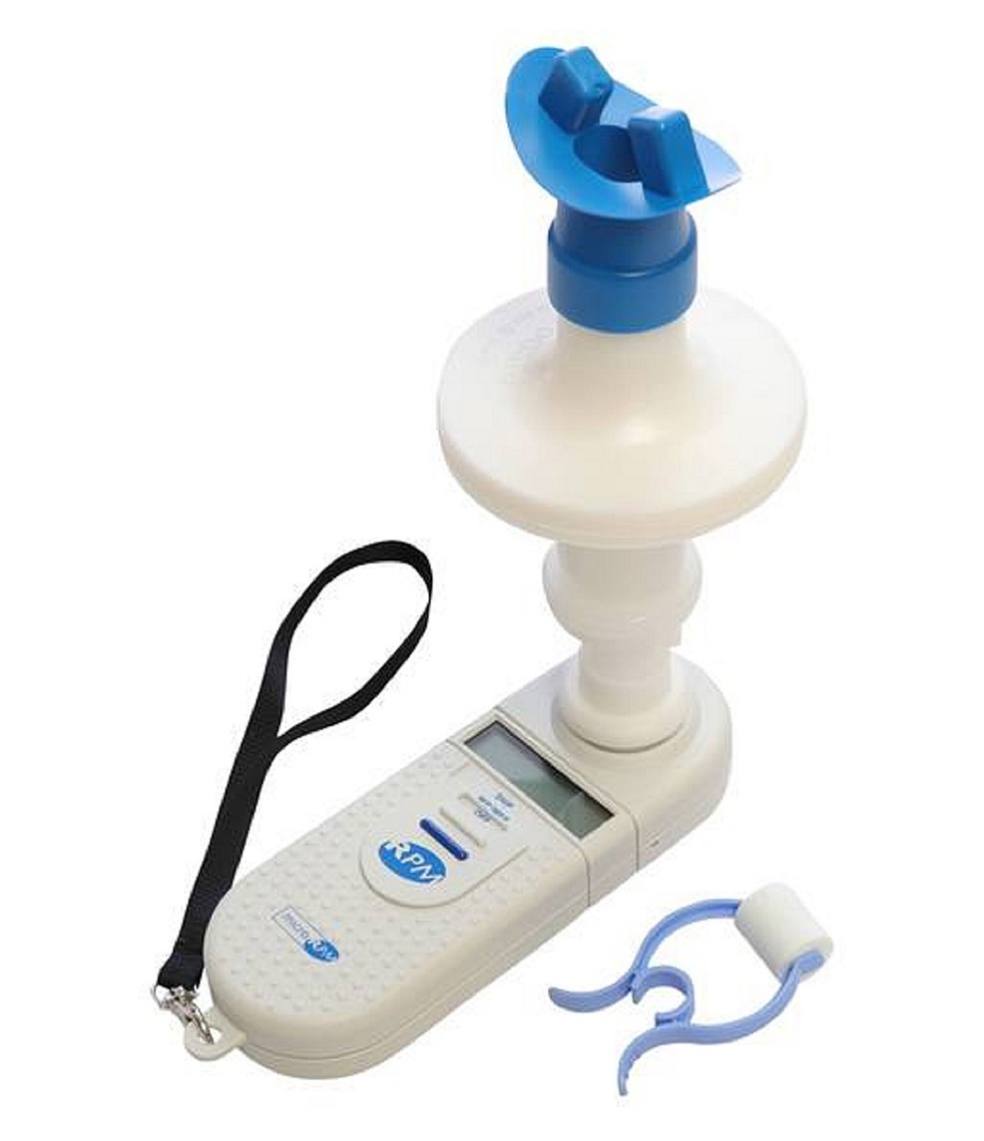
MicroRPM respiratory pressure meter.

The MicroRPM is a small, portable, lightweight, noninvasive, mouth-pressure manometer with a rubber-flanged mouthpiece (Figure 1). The MicroRPM displays the test results in a device monitor, uses software and calculates the MIP and MEP values, in cmH_^2^_O, from the one-second average maximum pressure [16].

AirOFit PRO™

**Figure 2:**
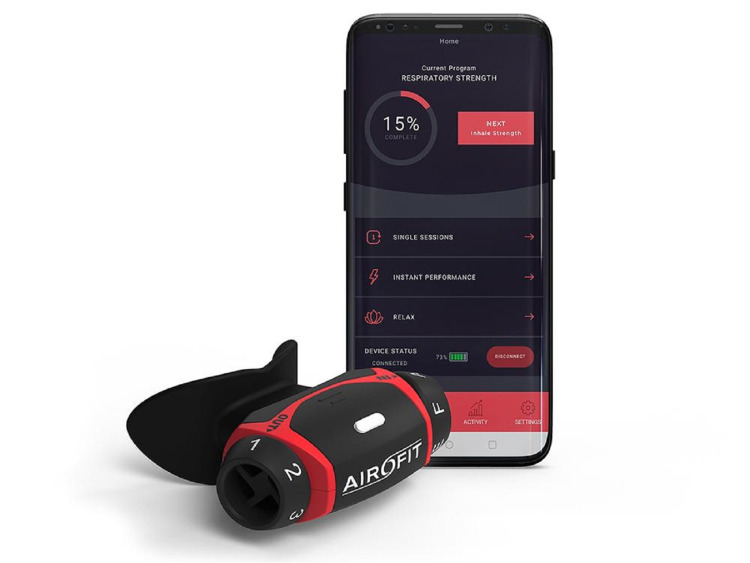
AirOFit PRO™ breathing trainer.

The AirOFit PRO™ is a small, portable, lightweight, noninvasive, mouth-pressure manometer with a rubber-flanged mouthpiece for assessment and training the respiratory muscles (Figure 2). AirOFit PRO™ E-unit contains pressure sensors and a Bluetooth transmitter. This allows to measure the breathing patterns and visualize them on the phone via the AirOFit PRO™ Sport mobile app. Moreover, the AirOFit PRO™ breathing trainer provides adjustable airflow resistance, making your respiratory muscles work overtime. Depending on the selected training program, duration and intensity, we are able to select the most appropriate resistance level. The AirOFit PRO™ generates resistance on respiratory muscles - primarily the diaphragm and the intercostal muscles, resulting in causing fatigue, which is then overcompensated by muscle tissue growth, making your breathing muscles faster, stronger and more efficient.

Procedures

For all athletes, the estimated strength of MIP and MEP were recorded by an AirOFit PRO™ and MicroRPM portable devices. The measurements (of both devices) were made in accordance with the ATS/ERS recommendation [12,17] and the manufacturer's instructions. All measurements were made in a quiet room, from a sitting position, and before the measurements, each participant performed six easy trials (3 × inspiratory and 3 × expiratory) as a warm-up and familiarity with the procedure. After 10 minutes, six maximum trials were made (3 × inspiratory and 3 × expiratory) with 45 seconds rest between efforts, and the largest one was recorded as the best trial. During measurements, all participants had closed lips firmly around the flanged mouthpiece and we applied a nose clip to avoid nasal air leak while the resting between trials (devices types) was 60 minutes. None of the participating athletes had previous experience in this test so that it does not exist learning effect. For each trial (MIP and MEP), we estimated the percentage of predicted values according to Wilson et al. formula [18]:

MIP (cmH_2_O) = 142 − (1.03 × age (years)) MEP (cmH_2_O) = 180 − (0.91 × age (years))

All participants after the efforts answered the question "easy-operation device-information" with scale 0-to-3 (0 = very bad, 1 = fairly bad, 2 = fairly good, 3 = very good) and dyspnea and/or respiratory fatigue during trials with CR10 scale [15]. All sessions were performed in the Laboratory of Cardio-Pulmonary Testing and Pulmonary Rehabilitation, (University of Thessaly), with the environmental temperature at 22±1 °C and humidity 45±3 %. The evaluation was made between 10:00 a.m. to 12:00 p.m. and all athletes did not have a previous exercise and/or training for 48 hours.

Statistical analysis

The Kolmogorov-Smirnov test was utilized to assess the normality of the distribution of values. Comparison of the same group of individuals to themselves (MicroRPM versus AirOFit PRO™ device) was performed with the Wilcoxon signed-rank test according to variable distribution. Bivariate correlation analysis was used for statistical comparison between parameters. Data are presented as absolute numbers, percentages or mean values and standard deviation (mean ±SD). For each test, the level of significance was set to p<0.05, and the data are presented as mean value and standard deviation (mean ± SD). The SPSS 25 statistical package (SPSS, Inc., Chicago, Illinois, USA) was used for the statistical analyses.

## Results

Respiratory parameters and ergospirometry results are presented in Table 1. Athletes, at the peak of ergospirometry, did not show exhaustion of breathing reserves (VE/MVV and TV/IC <85%, Table 1) and had a maximal oxygen uptake >100% of predicted. Correlations results showed statistical differences between VO_2max_ and maximal respiratory strength both for AirOFit PRO™ (r=0.526, p=0.014) and in MicroPRM (r=0.567, p=0.007).

The results of the PSQI questionnaire showed that according to their sleeping habits athletes are classified as good sleepers. The answers of athletes showed that they seemed to get enough sleep 8.2±1.2 hours per day, fell asleep quickly each night (10.6±4.0 min) and did not have problems during sleep, according to the PSQI score (1.9±1.8). The PSQI score showed statistical differences in % of predicted values in MEP with the AirOFit PRO™ device (r=0.478, p=0.028).

**Figure 3:**
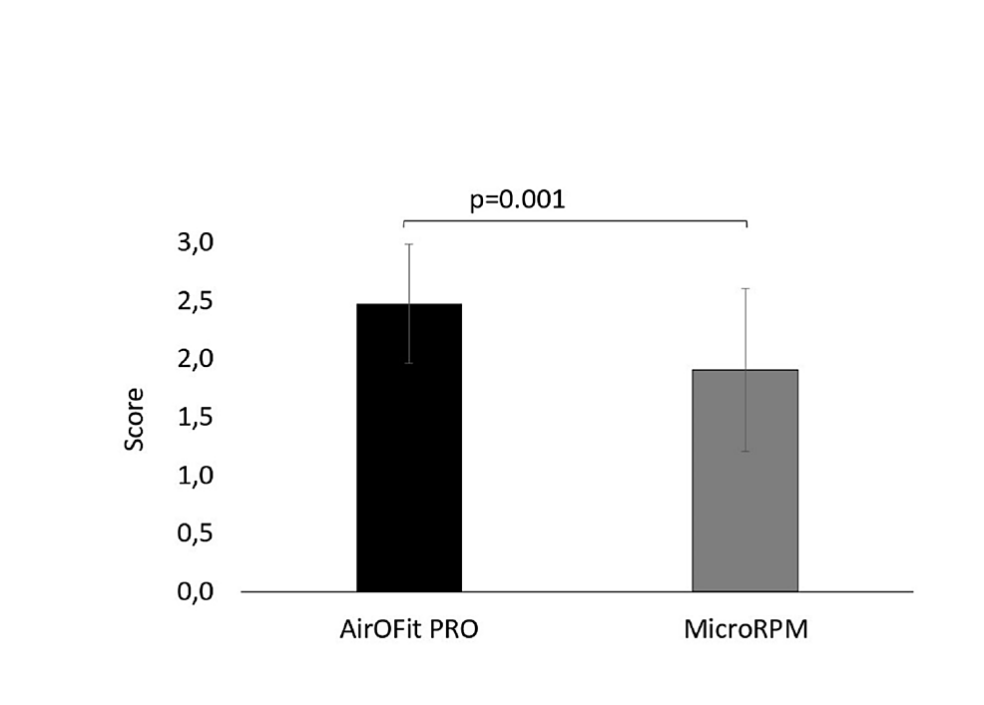
Results in question “easy-operation device-information” between trials.

**Figure 4:**
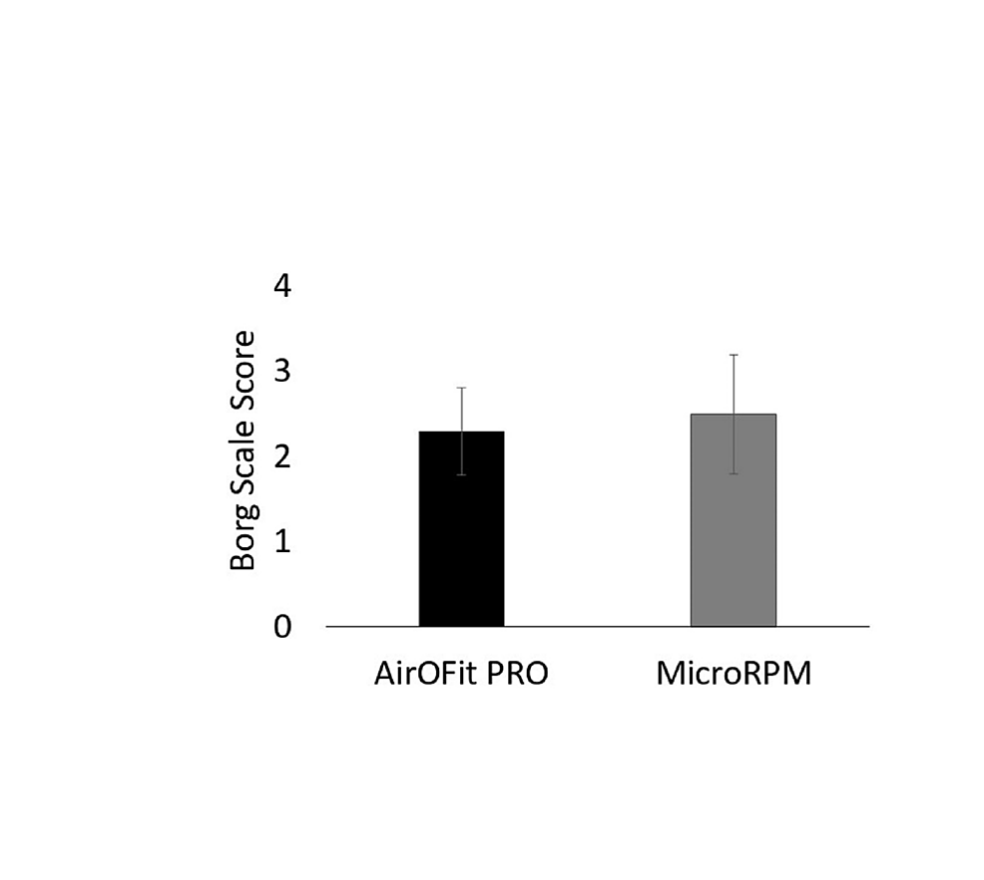
Results in question “dyspnea and/or respiratory fatigue” between trials.

Athletes did not show differences in RMS between devices (Table 2), while it was reported that the AirOFit PRO™ device is easier in operation as a device and provides more information during trial comparisons to MicroPRM (t(20)=3.873, p=0.001, Figure 3). Moreover, athletes did not report dyspnea and/or respiratory fatigue during trials in both devices (Figure 4).

**Table 2:** Respiratory muscle strength results. Continuous variables are presented as mean ± standard deviation. MEP: maximum expiratory pressure; MIP: maximum inspiratory pressure; RMS: respiratory muscle strength (MIP–MEP ratio); 95% Cl = 95% Confidence Interval of the difference.

	AirOFit PRO™	MicroPRM	95% Cl	P-value
MIP (cmH_^2^_O)	112.6±9.6	112.8±9.4	−2.509 to 2.128	0.866
MIP (% of predicted)	112.1±9.6	112.3±9.30	−2.583 to 2.226	0.879
MEP (cmH_^2^_O)	144.6±6.0	145.3±6.8	−1.702 to 0.274	0.147
MIP (% of predicted)	100.8±3.5	101.3±3.7	−1.168 to 0.202	0.157
RMS (cmH_^2^_O)	128.6±6.2	129.0±6.3	−1.834 to 0.929	0.503

## Discussion

Τhis study investigated the MIP and MEP, using two different portable devices in order to answer our hypotheses on the assessment of RMS and ease of use and information of each device.

The evaluation parameters between the two devices showed no differences in the MIP and MEP variables. On the contrary, statistically significant differences were observed between the devices in the parameter ease of use and information during the trials. The AirOFit PRO™ device seemed to be easier to use and provided more information to its users than the MicroPRM device due to its special software. The AirOFit PRO™ device, in addition to recording the strength of the respiratory muscles (MIP and MEP), has an application for the training of the respiratory muscles in order to improve respiratory function. According to its instructions, it has settings with resistors that can benefit the respiratory education of the general population, athletes and patients with chronic respiratory diseases. Specifically, the patient is given the opportunity to collect measurement data through the application, thus providing an overview of the total days of use of the device, while allowing immediate assessment of the level at which the patient - athlete is shown percentages of his measurements on the ideal and expected values based on the anthropometric characteristics of the patient. At the same time, with the AirOFit PRO™ device, there is the possibility for simultaneous inhalation and exhalation exercises, without the need to make any adjustments to the device, in contrast to the MicroRPM where a different adapter needs to be adjusted for inhalation and exhalation.

On the other hand, the MicroRPM device allows the usage by many patients with a short measurement interval, without the need for special preparation in terms of cleaning and disinfecting the device by simply adapting the respective inhaler and exhaler adapter and the removable mouthpiece compared to the AirOFit PRO™ device which has a built-in nozzle and needs disinfection and cleaning before reused by a different patient.

In our study, we did not do intervention of exercises of respiratory muscles to observe changes in the strength of the respiratory muscles. Previous studies in athletes have shown that the use of a respiratory exerciser can improve performance and reduce exercise fatigue [5] and shortness of breath [19]. In addition, respiratory muscle exercisers are widely used to improve respiratory capacity in patients with COPD [20] and patients with other respiratory diseases [21] to improve their function and quality of life of the patients. According to the bibliography, the development of muscle strength is achieved at an intensity of 50-80% of maximum effort [22] with supervised exercise in special centers for patient safety. It is important to have studies comparing respiratory muscle exercises at a specific intensity and not with a subjective feeling of difficulty [23] in both athletes and patients with respiratory diseases to adjust the intensity to the needs of each user.

## Conclusions

Patients with respiratory diseases are increasing every year with new respiratory infections appearing, e.g., SARS-CoV-2. Finally, there are geographical and financial difficulties, and people who want to train cannot move for their training and/or their exercise and possibly respiratory tele-exercise and/or tele-rehabilitation with the AirOFit PRO™ device would be an important and safe training solution. Proposal for future research will be to investigating whether the parameters of spirometry, RMS and cardiopulmonary exercise testing are affected by the AiroFit PRO™ breathing trainer.
